# Genetic Variance in the Spinocerebellar Ataxia Type 2 (*ATXN2*) Gene in Children with Severe Early Onset Obesity

**DOI:** 10.1371/journal.pone.0008280

**Published:** 2009-12-14

**Authors:** Karla P. Figueroa, Sadaf Farooqi, Kristopher Harrup, Johnathan Frank, Stephen O'Rahilly, Stefan M. Pulst

**Affiliations:** 1 Department of Neurology, University of Utah, Salt Lake City, Utah, United States of America; 2 Metabolic Research Laboratories, Institute of Metabolic Science, Addenbrooke's Hospital, University of Cambridge, Cambridge, United Kingdom; 3 David Geffen Medical School, University of California Los Angeles, Los Angeles, California, United States of America; 4 Program in Neurosciences and Brain Institute, University of Utah, Salt Lake City, Utah, United States of America; University of São Paulo, Brazil

## Abstract

**Background:**

Expansion of a CAG repeat in the coding region of exon 1 in the *ATXN2* gene located in human chromosome 12q24.1 causes the neurodegenerative disease spinocerebellar ataxia type 2 (SCA2). In contrast to other polyglutamine (polyQ) disorders, the SCA2 repeat is not highly polymorphic in central European (CEU) controls with Q22 representing 90% of alleles, and Q23 contributing between 5–7% of alleles. Recently, the *ATXN2* CAG repeat has been identified as a target of adaptive selection in the CEU population. Mouse lines deficient for atxn2 develop marked hyperphagia and obesity raising the possibility that loss-of-function mutations in the *ATXN2* gene may be related to energy balance in humans. Some linkage studies of obesity related phenotypes such as antipsychotic induced weight gain have reported significant lod scores on chromosome 12q24. We tested the hypothesis that rare loss-of-function *ATXN2* variants cause obesity analogous to rare mutations in the leptin, leptin receptor and MC4R genes.

**Methodology/Principal Findings:**

We sequenced the coding region of *ATXN2* including intron-exon boundaries in 92 severely obese children with a body mass index (BMI) >3.2 standard deviations above age- and gender-adjusted means. We confirmed five previously identified single nucleotide polymorphisms (SNPs) and three new SNPs resulting in two synonymous substitutions and one intronic polymorphism. Alleles encoding >Q22 were overrepresented in our sample of obese children and contributed 15% of alleles in children identified by their parents as white. SNP rs695872 closely flanking the CAG repeat showed a greatly increased frequency of C/C homozygotes and G/C heterozygotes compared with reported frequencies in the CEU population.

**Conclusions/Significance:**

Although we did not identify variants leading to novel amino acid substitutions, nonsense or frameshift mutations, this study warrants further examination of variation in the *ATXN2* gene in obesity and related phenotypes in a larger case-control study with emphasis on rs695872 and CAG repeat structure.

## Introduction

The ataxin-2 protein is a 1312 amino acid protein of unknown function encoded by 25 coding exons of the SCA2*/ATXN2* gene [1], [2]. The protein contains a polyglutamine domain, which is encoded by a CAG/CAA repeat in exon 1 of the gene. Expansion of the CAG repeat and the consequent elongation of the polyQ domain to ≥32 repeats causes the neurodegenerative disease spinocerebellar ataxia type 2 (SCA2). *ATXN2* is widely expressed in neuronal and non-neuronal tissues and is highly conserved in the mouse at the nucleotide and amino acid level [3], [4]. The region containing the *ATXN2* gene on 12q24.1 has undergone significant selection in Central Europeans (CEU) [5]. The selected allele is associated with a specific twice CAG repeat that is interrupted twice by a CAA codon.

The function of *ATXN2* has remained largely unknown. It is a cytoplasmic protein with Golgi association [6] which may play a role in RNA splicing via its interaction with A2BP1/FOX-1 [7], [8]. An association with polyribosomes has been observed in the fly [9]. Ralser and colleagues identified interactions between ataxin-2 and endophilin proteins in plastin-associated cellular pathways [10]. ATXN2 also interacts with the DEAD/H-box RNA helicase DDX6, components of P-bodies and stress granules[11].

*ATXN2* deficiency in the rodent did not result in a neurodegenerative phenotype, but led to marked obesity [12]. These results have recently found independent confirmation [13]. Genetic linkage studies of human obesity-related traits have also implicated the *ATXN2* region on human chromosome 12q24. Li and coworkers identified linkage for body mass index (BMI) and total fat percentage to D12S2070, a marker that is 4MB telomeric to the *ATXN2* gene[14]. This region has also been implicated in studies of obesity associated with antipsychotic medication. Highest lod scores were obtained with the marker D12S79, which is 4MB telomeric of *ATXN2* [*15*]. Parent-of-origin analysis of obesity traits implicated the region around D12S2070 as well with higher lod scores obtained for maternal transmissions [16]. Other groups, however, identified linkage signals further distal to the *ATXN2* locus in 12q24.3 [17], [18]. To elucidate a potential role for *ATXN2* in human obesity we examined the coding region of *ATXN2* in 92 patients with severe obesity of early onset recruited to the UK Genetics of Obesity Study.

## Results

Based on our finding of hyperphagia and obesity in mice hemizygously or homozygously deficient for *ATXN2* we tested the hypothesis that loss-of-function mutations in *ATXN2* are responsible for a subset of human obesity. We sequenced the 25 coding exons of the human *ATXN2* gene in 92 severely obese children enrolled in the Genetics of Obesity Study (GOOS) with a mean body mass index >3.2 standard deviations above the mean. Using primers located in the introns flanking the respective exon (Table S1) we searched for coding sequence variation as well as for variants leading to an alteration in splicing.

Table 1 lists the location and nature of eight DNA sequence variants separately for our entire cohort (n = 92) and those children with two self-identified white parents (n = 77) as well as SNP frequencies reported in the Hap-Map CEU population (n = 55). Overall, no variants were detected that changed the amino acid sequence of ataxin-2 other than one previously reported single nucleotide polymorphism (SNP). This SNP (rs695871) had the same genotype frequencies as reported for the CEU reference population (Table 1). We also identified two rare synonymous changes in exons 1 and 23 that had not previously been reported. These basepair changes are likely rare normal variants and are not expected to induce cryptic splice sites.

**Table 1:** *ATXN2* Sequence Variants found in the Genetics of Obesity Study (GOOS) Cohort.

Location	SNP ID	Type	Flanking Sequence	Sequence Variation Site	Amino acid change	Genotype Frequency Total cohort	Genotype Frequency White Only	Frequency Hap-Map-CEU
Exon 1		Coding	GCCCGG**G/C**TGGCGC	c.81G>C	p. =	C/C = 0.00	C/C = 0.00	NA
						C/G = 0.02	C/G = 0.02	
						G/G = 0.98	G/G = 0.98	
Exon 1	rs695871	Coding	GTCGTC**C/G**TCCTTC	c.319C>G	p.Leu107Val	C/C = 0.07	C/C = 0.08	C/C = 0.07
						C/G = 0.35	C/G = 0.32	C/G = 0.42
						G/G = 0.58	G/G = 0.60	G/G = 0.51
Exon 1	rs695872**	Coding	CGCCCG**C/T**GCGTCC	c.390C>T	p. =	C/C = 0.08*	C/C = 0.08	C/C = 0.00
						C/T = 0.36*	C/T = 0.34	C/T = 0.06
						T/T = 0.57*	T/T = 0.58	T/T = 0.94
Intron 15		Non-Coding	TAGACC**A/C**TCCTGT	c.2882+24C>A		C/C = 0.96	C/C = 0.96	NA
						C/A = 0.04	C/A = 0.04	
						A/A = 0.00	A/A = 0.00	
Intron 21	rs12301585	Non-coding	TGGAGG**T/G**TGTCAG	c.3371-46T>G		G/G = 0.00	G/G = 0.00	G/G = 0.00
						G/T = 0.02	G/T = 0.00	G/T = 0.00
						T/T = 0.98	T/T = 1.00	T/T = 1.00
Intron 21	rs2301622	Non-coding	TTGTCA**G/C**ACATCG	c.3371-40G>C		C/C = 0.08	C/C = 0.07	C/C = 0.03
						C/G = 0.40	C/G = 0.39	C/G = 0.41
						G/G = 0.52	G/G = 0.54	G/G = 0.55
Intron 22	rs2073950	Non-coding	TTTCCT**G/A**TCGCTT	c.3517-12G>A		A/A = 0.03	A/A = 0.04	A/A = 0.03
						A/G = 0.21*	A/G = 0.21	A/G = 0.42
						G/G = 0.76*	G/G = 0.75	G/G = 0.55
Exon 23		Coding	GCCGGC**G/A**TATACC	c.3744G>A	p. =	A/A = 0.00	A/A = 0.00	NA
						A/G = 0.04	A/G = 0.05	
						G/G = 0.96	G/G = 0.95	

**RefSeq NM_002973.3.**

*The genotype frequencies are statistically different than those reported for the Hap-Map CEU population.

**Frequencies for Hap-Map CEU not available for this SNP, CEPH frequencies given.

Two additional SNPs had been previously reported in European and Asian populations; rs695872 is located in exon 1 just upstream of the CAG repeat and results in a synonymous change, rs2073950 is located in intron 22. For both SNPs, genotype frequencies in the samples of obese white children from the U.K. were significantly different from those reported in the Hap-Map CEU sample (Table 1; rs695872, chi-square 36, DF 2, nominal p<0.0000001; rs2073950, chi-square 10.2, DF 2, nominal p<0.007). Both SNPs have significantly different allele frequencies in white and Asian populations.

We also determined the size and structure of the CAG/CAA repeat as the CAG repeat is known to be interrupted by CAA codons. This repeat is relatively invariant in the CEU population, but highly variable in Africans. The most common allele worldwide has 22 repeats with a composition of (CAG)_8_CAA(CAG)_4_CAA(CAG)_8_ and has undergone adaptive selection in the CEU population [5]. We confirmed the known repeat configurations in our sample and detected a total of 15 different CAG/CAA repeat alleles (Table 2, Table S2). Five of the alleles (CAG)_10_CAA(CAG)_11_; (CAG)_8_CAA(CAG) _5_CAA(CAG)_7_; (CAG)_25_; (CAG)_13_CAA(CAG)_11_; (CAG)_13_CAACAG_13_ ) had not been previously identified in over 900 alleles worldwide. Overall, we detected a higher frequency of alleles with 23 or more repeats in our white cohort (14.9%) than reported for the CEU (9.1%, n = 110) or the Polish (7.7%, n = 234) populations. Given the large numbers of comparisons necessary, we did not attempt a statistical analysis of CAG repeat allele frequencies and repeat alleles. A larger number of obese individuals need to be examined to determine whether particular repeat alleles are associated with the development of obesity.

**Table 2:** Allele Frequency in the GOOS Cohort and 9 other Populations.

Repeat Structure	Repeat size	Mixed Pop n = 184^a^	White Only* n = 154^a^	CEU n = 110^b^	French n = 17^c^	Spanish n = 11^d^	Polish n234^e^	Indian n = 215^f^	CHB n = 86^b^	JPT n = 129^b, g, h^	YRI n = 106^b^
(CAG)12CAA(CAG)8	21		0.006 (1)				0.004 (1)	0.005 (1)			
(CAG)8CAA(CAG)4CAA(CAG)7	21	0.005 (1)	0.006 (1)							0.023 (3)	
(CAG)22	22										0.009 (1)
(CAG)8CAA(CAG)13	22	0.005 (1)	0.006 (1)								0.009 (1)
(CAG)10CAA(CAG)11	22	0.005 (1)	0.006 (1)								
(CAG)13CAA(CAG)8	22	0.14 (26)	0.136 (21)	0.173 (19)	0.118 (2)		0.145 (34)	0.205 (44)	0.442 (38)	0.217 (28)	0.132 (14)
(CAG)8CAA(CAG)4CAA(CAG)8	22	0.696 (128)	0.688 (106)	0.727 (80)	0.529 (9)	0.455 (5)	0.765 (179)	0.707 (152)	0.558 (48)	0.744 (96)	0.358 (38)
(CAG)8CAA(CAG)5CAA(CAG)7	22	0.005 (1)									
(CAG)9CAA(CAG)4CAA(CAG)8	23	0.005 (1)	0.006 (1)				0.004 (1)	0.005 (1)			
(CAG)13CAA(CAG)9	23	0.043 (8)	0.052 (8)	0.009 (1)			0.021 (5)	0.014 (3)			
(CAG)14CAA(CAG)8	23	0.049 (9)	0.058 (9)	0.064 (7)			0.030 (7)	0.014 (3)			
(CAG)8CAA(CAG)14	23	0.005 (1)	0.006 (1)		0.059 (1)						0.028 (3)
(CAG)8CAA(CAG)5CAA(CAG)8	23	0.016 (3)			0.059 (1)						0.377 (40)
(CAG)25	25	0.011 (2)	0.013 (2)								
(CAG)13CAA(CAG)11	25	0.005 (1)	0.006 (1)								
(CAG)13CAA(CAG)13	27	0.005 (1)	0.006 (1)								
NA**	≥27			0.018 (2)	0.118 (2)	0.090 (2)	0.017 (4)	0.023 (5)			0.009 (1)

a Pulst et al (2009), ^b^ Gibbs et al (2005),^c^ Imbert et al (1996), ^d^ Pujana et al (1999),^ e^ Krzyzosiak et al (2004),^ f^ Choudhry et al, ^g^ Mizuhima et al (1999), ^h^ Sanpei et al (1996).

*This number is contained within the n = 184 of the Mixed population.

**Repeats ≥27 CAG repeats not found within our cohort.

## Discussion

The *ATXN2* gene was initially identified as the gene mutated in SCA2, a human neurodegenerative disease. The only mutations identified so far consist of expansion of a CAG repeat in exon 1, which encodes a polyglutamine domain leading to aggregation of the protein in neurons [3], [19], [20]. Knock-out of the *ATXN2* gene in the mouse resulted in marked obesity with onset shortly after weaning [12], [13]. Weight gain occurred as a result of increased food intake. This phenotype was also observed in heterozygote animals. Hyperphagia and weight gain were intermediate between wildtype and homozygote knockouts pointing to subtle effects of gene dosage (Pulst SM, unpublished).

Other types of mutations have not been described in humans and few coding variants have been deposited into genomic databases. The rationale for the present study was provided by the finding of obesity in *Atxn2* knockout mice [12] and the presence of linkage signals for obesity-related traits on 12q24, the location of the human *ATXN2* gene [14], [15], [16]. We hypothesized that we would detect rare loss-of-function genetic variants similar to the link between obesity and rare variants in the leptin, leptin receptor and MC4R genes [21].

We sequenced the 25 coding exons and intron-exon boundaries of *ATXN2* in children with severe obesity. Our results did not support a major role of *ATXN2* coding variants in the causation of human childhood obesity. The paucity of coding variants in this relatively large gene was surprising as we did not identify any amino acid substitutions other than the known p.Leu107Val (rs695871) polymorphism. Nonsense mutations, or changes near exon-intron boundaries were absent in the 92 samples, One synonymous coding SNP (rs695872), and one intronic SNP (rs2073950) showed nominally significant deviations from genotype frequencies reported for the CEU population sample. Our study was not designed as a case-control allelic association study and these observations need to be repeated in an ethnically matched sample of obese and lean children.

Recent studies suggested that evolutionary selection acted upon the *ATXN2* CAG repeat or closely linked SNPs[5]. An allele with the (CAG)_8_CAA(CAG)_4_CAA(CAG)_8_ configuration appears to have been selected in Northern Europeans. Although CAA interruptions do not change the amino acid sequence, they lead to branched structures at the DNA and RNA level *in vitro* [22]. We therefore analyzed not only the repeat length, but also the structure of the repeat with respect to the location and number of CAA interruptions (Table 2, Table 2S). As reported for virtually all populations, the allele with 22 repeats and two CAA interruptions made up the vast majority of alleles. We did not find unusually expanded repeat lengths in the obese children, but 5 repeat structures that had not been previously seen in over 900 alleles worldwide. Four of these had ≥23 repeats. Overall repeats ≥23 were more common in white obese children than previously reported for CEU and Polish populations. It is important to note that an effect of the *ATXN2* repeat structure on obesity-related traits would be difficult to detect using adjacent SNPs in genome-wide association studies due to the higher mutation frequency of trinucleotide repeats in human populations.

Several limitations of our study need to be recognized. A sample size of 92 severely obese children may not be sufficient to detect rare variants. Our sequencing strategy would not have detected the presence of larger insertions/deletions and did not examine regions that likely control expression of the *ATXN2* gene. There are large evolutionarily conserved regions both upstream of the coding region, but also in intron 1 of the *ATXN2* gene. These regions deserve further study before variants in the *ATXN2* gene can be excluded from contributing to variation in human body weight or related phenotypes. Furthermore, *ATXN2* gene variants could be important for other obesity-related phenotypes such as later-onset obesity or obesity associated with developmental delay. This study, however, provides a framework for the interpretation of future coding sequence variants and the possibility for understanding adaptive selection biases acting upon the *ATXN2* repeat structure or flanking regions.

## Materials and Methods

### Cohort

The Genetics of Obesity Study (GOOS) is a large cohort of patients with severe early onset obesity [23]. Inclusion criteria for the GOOS cohort are severe obesity defined as a BMI standard deviation score (S.D.S) >3.2, and onset of obesity before 10 years of age. Ninety-two patients in whom the known monogenic causes of obesity had been excluded by sequencing of the genes encoding LEP, LEPR, POMC and MC4R were selected for this study. 77 patients in this study were U.K. white, 6 were Indonesian, 2 Afro-Caribbean and the remainder 7 of mixed background. Mean BMI was >3.2 standard deviations above the mean (corrected for age and gender), age of onset of obesity was 4 years.

### Ethics Statement

All subjects gave their written informed consent and the study was approved by the Cambridge Local Regional Ethics Committee, U.K. The study was conducted according to the principles of the Helsinki Declaration.

### Sequence Analysis

PCR reactions were done under the following conditions: 50 ng (5 µL) of genomic DNA, 80 ng of each primer (Table S1), 10 mM dNTP mix (Roche), 1X (2 µL) of 10X Buffer (Qiagen), 1 unit (0.2 µL) of Hot Taq (Qiagen), 2 µL of Q solution (Qiagen) and ddH_2_0 up to 20 µL, utilizing step down PCR in which the initial denaturation of 95°C for 15′ was followed by 10 cycles consisting of: 95°C 1′, annealing of 5°C higher than optimal temperature listed on primer table for 30″, 72°C 1′30″ followed by 30 cycles of 95°C 1′, optimal annealing temperature as listed per each exon for 30″, 72°C 1′ 30″ followed by an extension of 72°C for 5′. Due to the large size of exon 1, it was amplified at 95°C for 15′, 35 cycles of 95°C 1′ 30″, 65°C 30 sec, 72°C 2 min with a final extension of 72′C for 7 min. PCR products were checked on agarose gels, and then sequenced as detailed below. Each fragment containing mutations was PCR amplified and sequenced a second time to confirm that the identified mutations were not due to PCR artifact.

DNA sequencing was performed using the ABI BigDye Terminator v3.1 cycle sequencing kit and the following protocol: 10 ng (2 µL) of purified PCR amplicon, 3 µL sequencing reaction pre-mix, 2 µL 5X sequencing buffer, 80 ng (2 µL) of primer and 11 µL of DD H20. The reaction mix was run in a PCR thermocycler (Bio-Rad MyCycler v 1.065) and cycled as follows: 96′C for 3 min followed by 25 cycles consisting of 96′C for 10 s, 50 for 5 s and 60′C for 4 min. Sequencing products were purified using ABI Centri-Sep spin columns. Resuspended samples were electrophoresed on an ABI 377 DNA sequencer. All sequences were analyzed using BioEdit biological sequence alignment editor (v5.0.9.1, Tom Hall, Isis Pharmaceuticals).

Sequencing of the CAG repeat: Primer labeling was set up with 25pmol of the SCA2-A (5′GGGCCCCTCACCATGTCG3′) oligonucleotide primer end-labeled with γ-^32^P ATP (3,000 Ci/nmol, 10 mCi/ml, 50 pmol total), 5 units (5 µL) of T4 Polynucleotide Kinase (Epicentre), 5 µL of 10X Kinase Buffer and ddH_2_0 up to 50 µL. This reaction mix was incubated at 37°C for 30 min then heat inactivated at 70°C for 5 min.

Sequencing took place with the same cycling conditions described above for exon 1. As template, we used 50–100fmol of exon 1 amplicon. The sequencing protocol was followed as described in the SequiTherm EXCEL II DNA sequencing kit. Reaction products were resolved in a standard 6% denaturing polyacrylamide gel, 8 M urea.

## Supporting Information

Table S1 SCA2 Primer Table and predicted amplicon sizes
(0.02 MB XLS)

Table S2 Comprehensive SCA2 CAG Repeat Structure and Frequency in 9 Populations
(0.02 MB PDF)
