# Supplementary material for: Genetic Variance in the Spinocerebellar Ataxia Type 2 (ATXN2) Gene in Children with Severe Early Onset Obesity
Source: PLoS One. 2009 Dec 14;4(12):e8280. doi: 10.1371/journal.pone.0008280 (PMC2791421; doi:10.1371/journal.pone.0008280)
Supplement: Table S2 — Comprehensive SCA2 CAG Repeat Structure and Frequency in 9 Populations (0.02 MB PDF) [file pone.0008280.s002.pdf]

Allele Frequency in 9 Populations (n)

Allele	Repeat Structure	Total repeat	Mixed Population (n=184)[a]	Caucasian Only* (n=154)[a]	CEU (n=110)[b]	French (n=17)[c]	Spanish (n=11)[d]	Polish (n234)[e]	Indian (n=215)[f]	CHB (n=86)[b]	JPT (n=129)[b, g, h]	YRI (n=106)[b]	Total in all populations (n=1092)
1	(CAG)8CAA(CAG)5	14						0.004 (1)					0.001 (1)
2	(CAG)6CAA(CAG)8	15									0.008 (1)		0.001 (1)
3	(CAG)8CAA(CAG)8	17				0.118 (2)	0.273 (3)						0.005 (5)
4	(CAG)9CAA(CAG)8	18							0.005 (1)				0.001 (1)
5	(CAG)13CAA(CAG)5	19						0.004 (1)			0.008 (1)		0.002 (2)
6	(CAG)10CAA(CAG)8	19			0.009 (1)								0.001 (1)
7	(CAG)12CAA(CAG)8	21		0.006 (1)				0.004 (1)	0.005 (1)				0.002 (2)
8	(CAG)8CAA(CAG)4CAA(CAG)7	21	0.005 (1)	0.006 (1)							0.023 (3)		0.004 (4)
9	(CAG)22	22										0.009 (1)	0.001 (1)
10	(CAG)8CAA(CAG)13	22	0.005 (1)	0.006 (1)								0.009 (1)	0.002 (2)
11	(CAG)10CAA(CAG)11	22	0.005 (1)	0.006 (1)									0.001 (1)
12	(CAG)13CAA(CAG)8	22	0.14 (26)	0.136 (21)	0.173 (19)	0.118 (2)		0.145 (34)	0.205 (44)	0.442 (38)	0.217 (28)	0.132 (14)	0.188 (205)
13	(CAG)8CAA(CAG)4CAA(CAG)8	22	0.696 (128)	0.688 (106)	0.727 (80)	0.529 (9)	0.455 (5)	0.765 (179)	0.707 (152)	0.558 (48)	0.744 (96)	0.358 (38)	0.673 (735)
14	(CAG)8CAA(CAG)5CAA(CAG)7	22	0.005 (1)										0.001 (1)
15	(CAG)23	23							0.005 (1)			0.019 (2)	0.003 (3)
16	(CAG)9CAA(CAG)4CAA(CAG)8	23	0.005 (1)	0.006 (1)				0.004 (1)	0.005 (1)				0.003 (3)
17	(CAG)13CAA(CAG)9	23	0.043 (8)	0.052 (8)	0.009 (1)			0.021 (5)	0.014 (3)				0.016 (17)
18	(CAG)14CAA(CAG)8	23	0.049 (9)	0.058 (9)	0.064 (7)			0.030 (7)	0.014 (3)				0.024 (26)
19	(CAG)8CAA(CAG)14	23	0.005 (1)	0.006 (1)		0.059 (1)						0.028 (3)	0.005 (5)
20	(CAG)8CAA(CAG)5CAA(CAG)8	23	0.016 (3)			0.059 (1)						0.377 (40)	0.040 (44)
21	(CAG)8CAA(CAG)4CAA(CAG)9	23							0.009 (2)				0.002 (2)
22	(CAG)24	24							0.009 (2)				0.002 (2)
23	(CAG)15CAA(CAG)8	24						0.004 (1)				0.038 (4)	0.004 (5)
24	(CAG)25	25	0.011 (2)	0.013 (2)									0.002 (2)
25	(CAG)13CAA(CAG)11	25	0.005 (1)	0.006 (1)									0.001 (1)
26	(CAG)15CAA(CAG)9	25					0.091 (1)						0.001 (1)
27	(CAG)16CAA(CAG)8	25										0.009 (1)	0.001 (1)
28	(CAG)13CAA(CAG)2CAA(CAG)8	25										0.009 (1)	0.001 (1)
29	(CAG)13CAA(CAG)13	27	0.005 (1)	0.006 (1)									0.002 (1)
30	(CAG)8CAA(CAG)4CAA(CAG)4CAA(CAG)8	27			0.018 (2)	0.059 (1)	0.091 (1)	0.013 (3)					0.006 (7)
31	(CAG)12CAA(CAG)5CAA(CAG)9	28				0.059 (1)							0.001 (1)
32	(CAG)29	29							0.005 (1)				0.001 (1)
33	(CAG)8CAA(CAG)4CAA(CAG)4CAA(CAG)10	29					0.091 (1)	0.004 (1)	0.005 (1)				0.003 (3)
34	(CAG)8CAA(CAG)5CAA(CAG)5CAA(CAG)8	29										0.009 (1)	0.001 (1)
35	(CAG)20CAA(CAG)8	29							0.005 (1)				0.001 (1)
36	(CAG)13CAA(CAG)7CAA(CAG)8	30							0.009 (2)				0.002 (2)

[a] Pulst et al (2007), [b] Gibbs et al (2005),[c] Imbert et al (1996), [d] Pujana et al (1999), [e] Krzyzosiak et al (2004), [f] Choudhry et al, [g] Mizuhima et al (1999), [h] Sanpei et al (1996)

* This number is contained within the n=184 of the Mixed population
